# *Lonicerae Japonicae Caulis*: a review of its research progress of active metabolites and pharmacological effects

**DOI:** 10.3389/fphar.2023.1277283

**Published:** 2023-10-25

**Authors:** Yu-Xia Cao, Peng Ji, Fan-Lin Wu, Jia-Qi Dong, Chen-Chen Li, Ting Ma, Hao-Chi Yang, Yan-Ming Wei, Yong-Li Hua

**Affiliations:** College of Veterinary Medicine, Gansu Agricultural University, Lanzhou, Gansu, China

**Keywords:** *Lonicerae Japonicae Caulis*, active metabolites, pharmacological effects, mechanism of action, research progress

## Abstract

*Lonicerae Japonicae Caulis* is the aboveground stem part of the *Lonicera Japonica* Thunb, which belongs to the medicine food homology species in China. It has the effects of clearing away heat, toxic material, dredging wind and unblocking collaterals. Modern research shows that it contains various active metabolites and a wide range of pharmacological effects, which is of great research and clinical application value. It mainly contains organic acids, volatile oils, flavonoids, triterpenes, triterpene saponins and other active metabolites. Its pharmacological effects mainly include anti-inflammatory, antibacterial, antitumor, antioxidant, and repairing bone and soft tissue. Based on the literature reports in recent years, the active metabolites, pharmacological effects and mechanisms of *Lonicerae Japonicae Caulis* were sorted out and summarized. It lays a foundation for explaining the efficacy material basis and application value of *Lonicerae Japonicae Caulis*. It aims to provide a reference for the in-depth research, development and utilization of *Lonicerae Japonicae Caulis*.

## 1 Introduction

*Lonicerae Japonicae Caulis* is also known as *Laowengxu*, *Salicylus vine*, *Psychic grass*, *Qianjin vine,* etc. It is a medicinal and food homologous Chinese medicinal material. Traditional Chinese medicine theory believes that its nature is sweet flavor and cold. It has been mainly treated for warm-disease fever, sore carbuncle and swollen toxin, heat toxin and blood dysentery, beriberi pyretic arthralgia, arthralgia and myalgia, etc. It was initially published in the *Famous Doctors* and recorded in the *Compendium of Materia Medica*. The Chinese (veterinary) medical theory believed that *Lonicerae Japonicae Caulis* had effects of clearing away heat, toxic material, dredging wind and unblocking collaterals. Traditional Chinese medicine health cultivation known as *Medicine comes from food, food has medicinal properties, and medicine has food habits*. With the change in lifestyle and diet structure, it has entered the public’s field of vision and attracted widespread attention. *Lonicerae Japonicae Caulis*, a medicinal and edible dual-use Chinese medicinal material, has significant advantages in anti-inflammatory, antioxidant, antitumor and inhibition of pathogenic microorganisms and other aspects. In clinical practice, it can to treat acute fever, headache, sore throat, respiratory infections and arthritis (Li and Mu, 2017). *Lonicerae Japonicae Caulis* contains organic acids, volatile oils, flavonoids, triterpenes, triterpene saponins and other active metabolites, of which chlorogenic acid (CGA) is one of the characteristic active metabolites of *Lonicerae Japonicae Caulis*. By reviewing the literature on *Lonicerae Japonicae Caulis* in recent years, this paper reviews and analyzes the active metabolites, pharmacological effects and mechanisms of *Lonicerae Japonicae Caulis.* It aims to provide a reference for the subsequent research, development and utilization of *Lonicerae Japonicae Caulis* and to look forward to its research prospects.

## 2 Main active metabolites of *Lonicerae Japonicae Caulis*

The main active metabolites of *Lonicerae Japonicae Caulis* include organic acids, volatile oils, flavonoids and triterpenes, triterpene saponins, etc.

### 2.1 Organic acids

**FIGURE 1:**
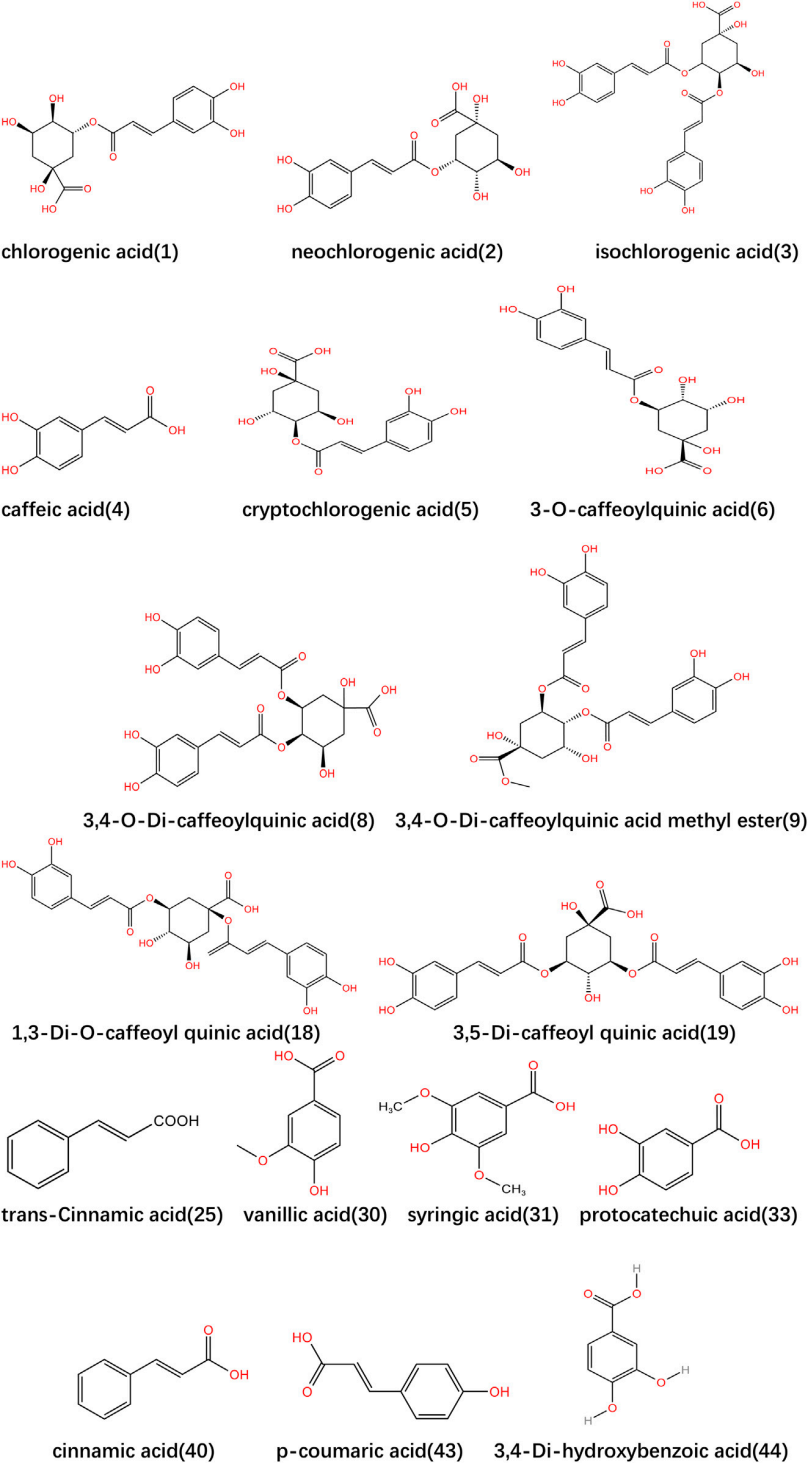
Structural formula of major organic compounds in *Lonicerae Japonicae (Caulis)*.

*Lonicerae Japonicae Caulis* is rich in organic acid metabolites, mainly including chlorogenic acid derivatives and cinnamic acid derivatives. Chlorogenic acid derivatives mainly include chlorogenic acid (CGA) and neochlorogenic acid, etc. (Lee et al., 2009; Lu, 2012; Seo et al., 2012; Yang et al., 2015; Li et al., 2019; Liu et al., 2020; Wang et al., 2020; Qiu et al., 2021). Cinnamic acid derivatives mainly include caffeic acid (CA), 1-caffeoylquinic acid (cryptochlorogenic acid), trans-cinnamic acid, trans-ferulic acid, and methyl caffeate (Li et al., 2019; Yang et al., 2015; Choi et al., 2007; Jeong et al., 2015). In terms of the inhibitory effect on human platelet aggregation, caffeic acid methyl ester, 3,4-di-O-caffeoyl quinic acid and 3,4-di-O-caffeoyl quinic acid methyl ester have strong effects. In addition, the protective effect of various phenolic acids on hydrogen peroxide-induced cell damage suggests that polyphenol metabolites may play a role in maintaining vascular homeostasis. Among them, chlorogenic acid and caffeic acid are the phenolic acids that are more studied at present, which belong to the essential active metabolites in *Lonicerae Japonicae Caulis*, and have anti-inflammatory and antioxidant effects at the same time (Hsu et al., 2016; Hou et al., 2017; Li N. et al., 2020). In addition, chlorogenic acid also has significant antitumor and hepatoprotective effects and is one of the most abundant organic acids in *Lonicerae Japonicae (Caulis)* (Li et al., 2019; Zhang et al., 2016; Yu et al., 2013; Tzeng et al., 2014; Chen et al., 2017). There is a high correlation between total phenolic content and antioxidant activity and phenolic metabolites have a significant contribution to the antioxidant capacity of traditional Chinese medicine. The organic acid content in *Lonicerae Japonicae Caulis* can be analyzed and determined with the Waters Symmetry C18 column (Tian et al., 2018). However, when establishing quantitative analysis of multi-metabolites by single marker (QAMS), there are differences in the mixing method, mixing position, and accuracy of mobile phases among different liquid chromatography systems, which may lead to differences in peak positioning. Therefore, when establishing QAMS, it is necessary to explore a more efficient and accurate approach. The specific information is shown in Table 1 and the structures of the main organic acid metabolites are shown in Figure 1.

**TABLE 1:** Composition of organic acid compounds in *Lonicerae Japonicae (Caulis)*.

No.	Compound Name	Formula	Ref.
1	chlorogenic acid	$C_{16}H_{18}O_{9}$	(Ma et al., 2009a)
2	neochlorogenic acid	$C_{16}H_{18}O_{9}$	(Wang et al., 2016)
3	isochlorogenic acid C	$C_{25}H_{24}O_{12}$	(Wang et al., 2016)
4	caffeic acid	$C_{9}H_{8}O_{4}$	(Zhang et al., 2009)
5	cryptochlorogenic acid	$C_{16}H_{18}O_{9}$	(Wang et al., 2016)
6	3-O-caffeoylquinic acid	$C_{16}H_{18}O_{9}$	(Wang et al., 2016)
7	4-O-caffeoylquinic acid	$C_{16}H_{18}O_{9}$	(Wang et al., 2016)
8	3,4-Di-O-caffeoylquinic acid	$C_{25}H_{24}O_{12}$	(Ma et al., 2009b)
9	3,4-Di-O-caffeoylquinic acid methyl ester	$C_{26}H_{26}O_{12}$	(Ma et al., 2009a)
10	ethyl 3,4-Di-O-caffeoylquinine	$C_{27}H_{28}O_{12}$	(Ma et al., 2009b)
11	3-O-caffeoylquinic acid methyl ester	$C_{17}H_{20}O_{9}$	(Wang et al., 2016)
12	3-O-caffeoylquinic acid ethyl ester	$C_{16}H_{18}O_{9}$	(Wang et al., 2016)
13	4-O-E-caffeoylquinic acid methyl ester	$C_{17}H_{20}O_{9}$	(Wang et al., 2016)
14	4,5-Di-O-caffeoylquinic acid	$C_{25}H_{24}O_{12}$	(Wang et al., 2016)
15	4,5-Di-O-caffeoylquinic acid methyl ester	$C_{26}H_{26}O_{12}$	(Wang et al., 2016)
16	5-O-caffeoylquinic acid methyl ester	$C_{25}H_{28}N_{2}O_{7}$	(Ma et al., 2009b)
17	5-O-caffeioyl quinine butyl ester	$C_{20}H_{26}O_{9}$	(Wang et al., 2016)
18	1,3-Di-O-caffeoylquinic acid	$C_{25}H_{24}O_{12}$	(Ma et al., 2009b)
19	3,5-Di-caffeoylquinic acid	$C_{25}H_{24}O_{12}$	(Liu and Chen, 2010)
20	3,5-Di-caffeioyl quinine butyl ester	$C_{28}H_{30}O_{12}$	(Wang et al., 2016)
21	ethyl 3,5-Di-caffeoylquinate	$C_{27}H_{28}O_{12}$	(Wang et al., 2016)
22	1,5-Di-caffeoylquinic acid	$C_{25}H_{24}O_{12}$	(Liu and Chen, 2010)
23	3,4,5-O-triformylquinic acid	$C_{34}H_{30}O_{15}$	(Wang et al., 2016)
24	caffeic acid-4-O- β- D-glucoside	$C_{15}H_{18}O_{9}$	(Ma et al., 2010)
25	trans-Cinnamic acid	$C_{9}H_{8}O_{2}$	(Wang et al., 2016)
26	ferulic acid	$C_{10}H_{10}O_{4}$	(Jia et al., 2015a)
27	trans-ferulic acid	$C_{10}H_{10}O_{4}$	(Wang et al., 2016)
28	caffeic acid methyl ester	$C_{10}H_{10}O_{4}$	(Jia et al., 2015b)
29	ethyl caffeate	$C_{11}H_{12}O_{4}$	(Jia et al., 2015a)
30	vanillic acid	$C_{8}H_{8}O_{4}$	(Wang et al., 2016)
31	syringic acid	$C_{9}H_{10}O_{5}$	(Wang et al., 2016)
32	loganic acid	$C_{16}H_{24}0_{10}$	(Wang et al., 2016)
33	protocatechuic acid	$C_{7}H_{6}0_{4}$	(Zhang et al., 2009)
34	cinerea caprifolia G	$C_{26}H_{26}O_{12}$	(Zhang et al., 2009)
35	methyl p-hydroxycinnamate	$C_{10}H_{10}O_{3}$	(Jia et al., 2015b)
36	ethyl laurate	$C_{14}H_{28}O_{2}$	(Wang et al., 2016)
37	2(E)-3-ethoxyacrylic acid	$C_{5}H_{8}O_{3}$	(Wang et al., 2016)
38	abscisic acid	$C_{15}H_{20}O_{4}$	(Wang et al., 2016)
39	3-(3,4-Di-hydroxyphenyl) propionic acid	$C_{9}H_{10}O_{4}$	(Wang et al., 2016)
40	cinnamic acid	$C_{9}H_{8}O_{2}$	(Wang et al., 2016)
41	4-hydroxycinnamic acid	$C_{9}H_{8}O_{3}$	(Wang et al., 2016)
42	methyl 4-hydroxycinnamate	$C_{10}H_{10}O_{3}$	(Wang et al., 2016)
43	p-coumaric acid	$C_{9}H_{8}O_{3}$	(Zhang et al., 2009)
44	3,4-Di-hydroxybenzoic acid	$C_{7}H_{6}O_{4}$	(Zhang et al., 2009)

### 2.2 Volatile oils

**FIGURE 2:**
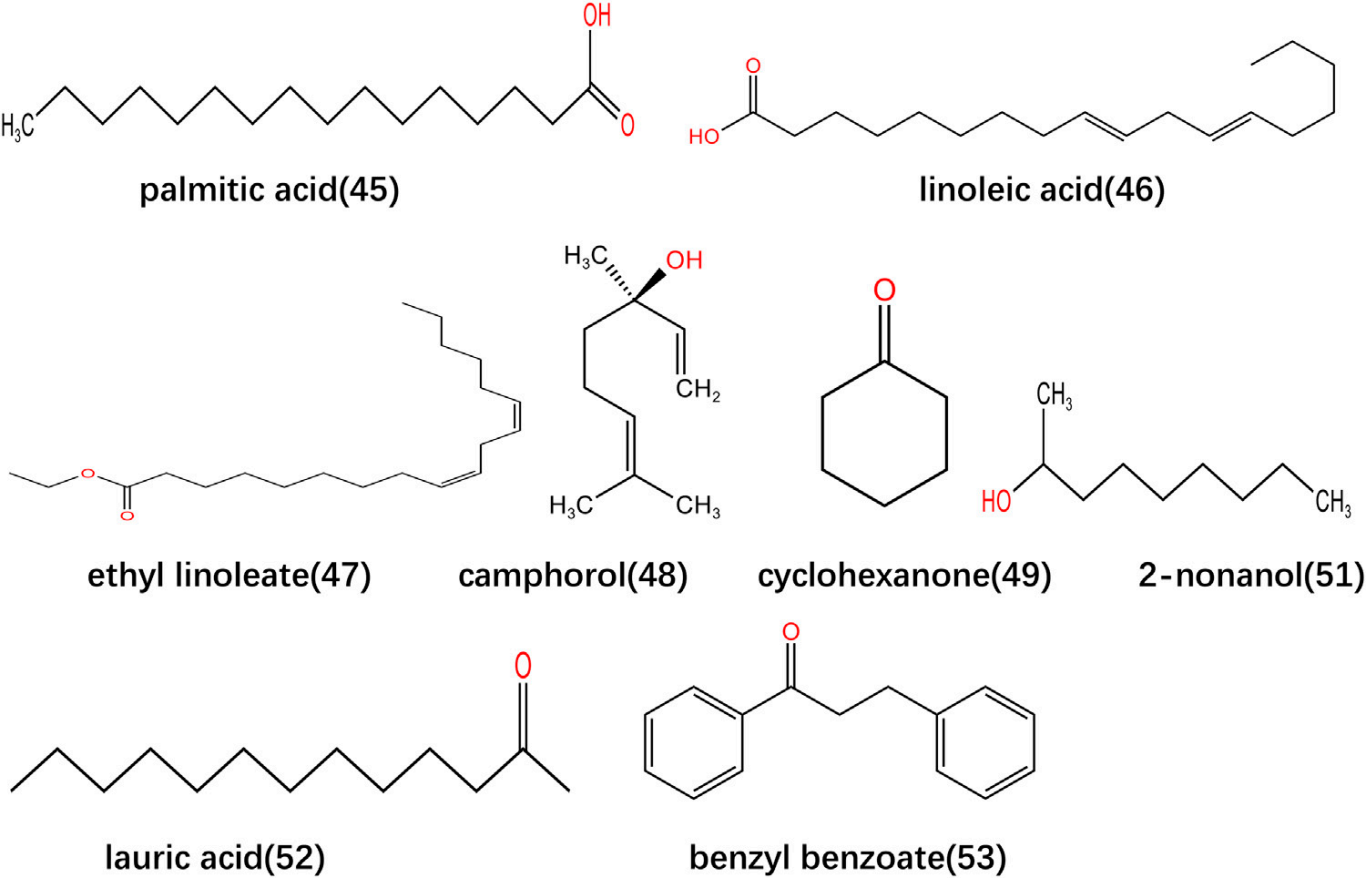
Structure of the main volatile oil compounds in *Lonicerae Japonicae (Caulis)*.

The volatile oils are the main active metabolites of *Lonicerae Japonicae*, mainly including acids, aldehydes, alcohols, ketones and their esters, which are present in the flowers, leaves and stems of *Lonicerae Japonicae*. In addition, it should be noted that different parts contain different volatile oil metabolites (Shang et al., 2011). Li Huijun isolated and identified the volatile oil metabolites in Lonicerae Japonicae Caulis, and confirmed that palmitic and linoleic acid had the highest content (Li et al., 2002). Wang Shuyan found that esters are the highest content of *Lonicerae Japonicae Caulis* volatile oil (Wang et al., 2011). Nenad Vukovic prepared essential oils from different parts of *Lonicerae Japonicae* and then carried out metabolite identification research with gas chromatography and mass spectrometry to. As a result, the researcher found that linalool was the primary volatile oil metabolite in honeysuckle flower buds, palmitic acid and linalool in honeysuckle leaves were the main volatile oil metabolites and palmitic acid in the stem was the primary volatile oil metabolite (Vukovic et al., 2012). However, there are also differences in the types and metabolites of volatile oils contained in *Lonicerae Japonicae (Caulis)* from different regions (Du et al., 2015). At present, qualitative and quantitative analysis among various studies shows that the metabolite composition of plants is greatly influenced by the environment and sample state. Therefore, when determining the content of metabolites in plants, it is necessary to consider the conditions of origin and the state of plant samples. The volatile oil in *Lonicerae Japonicae (Caulis)* is an edible natural flavour, which is currently mainly used in cosmetics, cigarettes, spices and food industries (Seo et al., 2012; Fang et al., 2020; Zhang et al., 2020). The volatile oils have certain activities in antibacterial, anti-inflammatory, and anti-tumor aspects. The main metabolites in the volatile oils, such as linolenic acid, palmitic acid and oleic acid, can affect the inflammatory factors nitric oxide (NO) and tumor necrosis factor (TNF)-α. And then inhibit the proliferation of breast cancer MCF-7 cells. In addition, it can effectively alleviate the occurrence of breast cancer and the inflammatory response in the early and late stages (Yang et al., 2018). Guo Fengyu et al. found that linalool accumulated on the cell membrane due to hydrophobic interactions. This phenomenon will cause the surface of the cell membrane to shrink, cause the formation of pores in the cell membrane and wall and lead to the leakage of small molecules. Therefore, it interferes with the regular operation of the ATP generation system inside bacteria and inhibits cell functional characteristics. And as the action time prolongs, the degree of damage increases with the increase in drug concentration (Guo et al., 2020). The specific information is shown in Table 2 and the structures of the main volatile oils metabolites are shown in Figure 2.

**TABLE 2:** Volatile oil compound components in *Lonicerae Japonicae (Caulis)*.

No.	Compound Name	Formula	Ref.
45	palmitic acid	$C_{16}H_{32}O_{2}$	(Li et al., 2002)
46	linoleic acid	$C_{18}H_{32}O_{2}$	(Li et al., 2002)
47	ethyl linoleate	$C_{20}H_{36}O_{2}$	(Li et al., 2002)
48	linalool	$C_{10}H_{18}O$	(Li et al., 2002)
49	cyclohexanone	$C_{6}H_{10}O$	(Wang et al., 2016)
50	cyclohexane	$C_{6}H_{12}$	(Wang et al., 2016)
51	2-Nonanol	$C_{9}H_{20}O$	(Wang et al., 2016)
52	lauric acid	$C_{12}H_{24}O_{2}$	(Wang et al., 2016)
53	benzyl benzoate	$C_{14}H_{12}O_{2}$	(Wang et al., 2016)
54	undecane	$C_{11}H_{24}$	(Wang et al., 2016)
55	tridecanal	$C_{13}H_{26}O$	(Wang et al., 2016)
56	tetradecanal	$C_{14}H_{28}O$	(Wang et al., 2016)
57	pentadecanal	$C_{15}H_{30}O$	(Wang et al., 2016)
58	myristate	$C_{14}H_{28}O_{2}$	(Wang et al., 2016)
59	pentadecanoic acid	$C_{15}H_{30}O_{2}$	(Wang et al., 2016)
60	cetyl alcohol	$C_{16}H_{34}O$	(Wang et al., 2016)
61	margaric acid	$C_{17}H_{34}O_{2}$	(Wang et al., 2016)

### 2.3 Flavonoids

**FIGURE 3:**
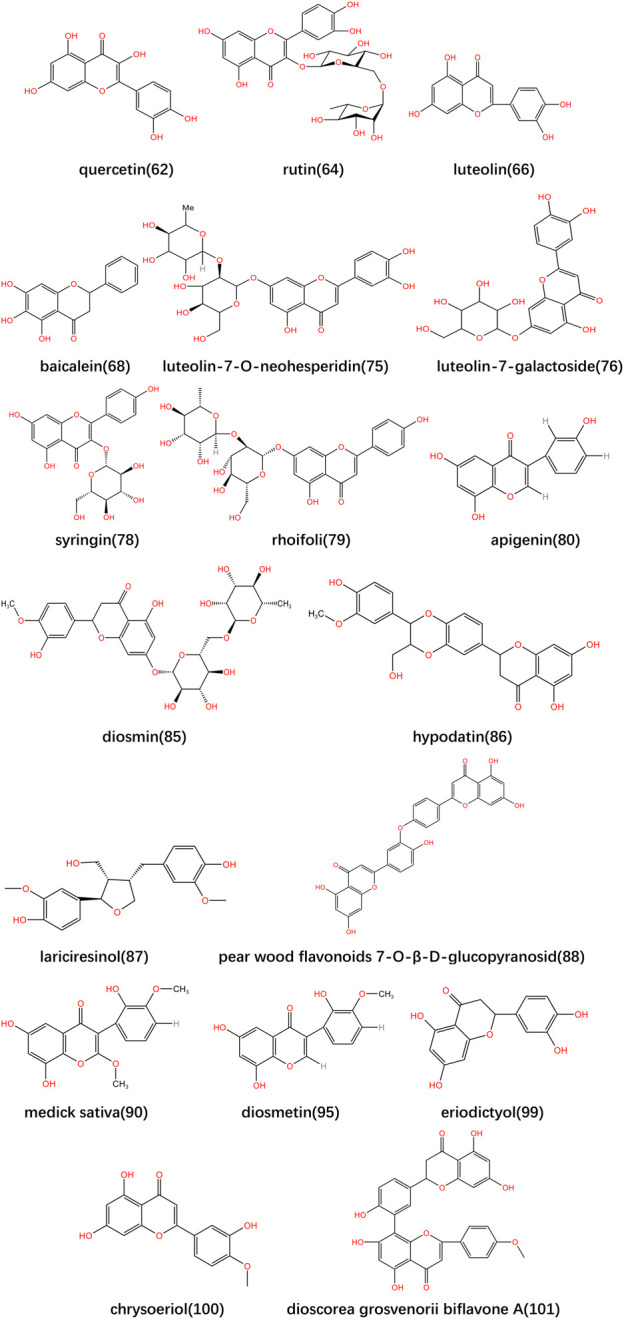
Structural formula of major flavonoids in *Lonicerae Japonicae (Caulis)*.

The flavonoids are the secondary metabolites prevalent in many natural plants (Han et al., 2016; Li N. et al., 2020; Liu et al., 2020). The structure is diversified, which include the circular matrix structure and O-and-C-glycosylated derivatives (Rauter et al., 2018). Currently, the flavonoids isolated from *Lonicerae Japonicae* can be divided into two categories: flavonoids and flavonols (Li et al., 2019). Among them, flavonoids include cinaroside, luteolin, witch hazel 7-O-neohesperidin, witch hazel 7-O-glucoside, cloxacillin and traxine, etc. (Choi et al., 2007; Lee et al., 2009; Fang et al., 2020). Flavonols mainly include rutin, quercetin, isoquercetin, baicalein and quercetin 3-O-hexosideetc (Lee et al., 2009; Seo et al., 2012). The specific information is shown in Table 3. The main pharmacological effects include antioxidant, bacteriostatic and antitumor activities. The flavonoids not only have an excellent preventive effect on cancer, but also have a certain effect on chronic diseases such as diabetes, cardiovascular and cerebrovascular diseases and liver diseases, etc. Among them, luteolin has anti-angiogenic effects on retinal microvessels, positive effects on inhibiting retinopathy in premature infants, and anti-inflammatory activity. With its ability to inhibit the oxidation of low-density lipoprotein, flavonoids exhibit unique cardioprotective effects and have a certain role in the prevention of cardiovascular diseases (Bagli et al., 2004; Ramassamy, 2006; Scalbert et al., 2007; Park et al., 2012; Han et al., 2016; Hsu et al., 2016; Hou et al., 2017; Ge L. L. et al., 2018; Wan et al., 2019; Li R. J. et al., 2020). The main metabolite types are shown in Table 3; The metabolites structures are shown in Figure 3.

**TABLE 3:** Flavonoid compound components in *Lonicerae Japonicae (Caulis)*.

No.	Compound Name	Formula	Ref.
62	quercetin	$C_{15}H_{10}O_{7}$	(Zhang et al., 2012)
63	isoquercetin	$C_{15}H_{10}O_{7}$	(Wang et al., 2016)
64	rutin	$C_{27}H_{30}O_{16}$	(Zhang et al., 2012)
65	naroside	$C_{35}H_{60}O_{6}$	(Wang et al., 2016)
66	luteolin	$C_{15}H_{10}O_{6}$	(Jia et al., 2015a)
67	tricine	————	(Wang et al., 2016)
68	baicalein	$C_{15}H_{10}O_{5}$	(Wang et al., 2016)
69	9-α-Hydroxy terpineol	————	(Shang et al., 2011)
70	quercetin 3-O-α-L-arabinopyranoside	————	(Shang et al., 2011)
71	quercetin-7-O-β-D-glucopyranoside	$C_{21}H_{20}O_{12}$	(Ma et al., 2010)
72	quercetin 3-O-β-D-glucopyranoside	————	(Shang et al., 2011)
73	chrysanthemulin	$C_{15}H_{10}O_{5}$	(Jia et al., 2015b)
74	3-methoxycarvacrol	————	(Jia et al., 2015a)
75	luteolin-7-O-neohesperidin	$C_{27}H_{30}O_{15}$	(Jia et al., 2015b)
76	luteolin-7-galactoside	————	(Jia et al., 2015a)
77	nivolumab-6-O-neohesperidin	————	(Jia et al., 2015b)
78	syringin	$C_{21}H_{20}O_{11}$	(Jia et al., 2015a)
79	rhoifolin	$C_{27}H_{30}O_{14}$	(Jia et al., 2015b)
80	apigenin	$C_{15}H_{10}O_{5}$	(Zhang et al., 2009)
81	luteolin-7-O- β- D-glucopyranoside	$C_{21}H_{20}O_{11}$	(Zhang et al., 2009)
82	isorhamnetin-7-O-β-D-glucopyranoside	$C_{22}H_{22}O_{12}$	(Zhang et al., 2009)
83	geranylgeranyl-7-O-β- D-glucopyranoside	$C_{22}H_{22}O_{11}$	(Zhang et al., 2009)
84	flavonoid lignans hydrocarpin D	————	(Zhang et al., 2009)
85	diosmin	$C_{28}H_{32}O_{15}$	(Ma et al., 2010)
86	hypodatin	$C_{25}H_{20}O_{9}$	(Ma et al., 2009a)
87	lariciresinol	$C_{20}H_{24}O_{6}$	(Shang et al., 2011)
88	pear wood flavonoids 7-O-β-D-glucopyranoside	$C_{30}H_{18}O_{10}$	(Ma et al., 2009b)
89	pear wood flavonoids	$C_{30}H_{18}O_{10}$	(Ma et al., 2009a)
90	medicksativa	$C_{17}H_{14}O_{7}$	(Ma et al., 2009b)
91	5,7,4 '- trihydroxy-8-methoxyflavonoid	————	(Ma et al., 2009a)
92	naphthol 7-O-β-D-glucopyranoside	$C_{21}H_{20}O_{11}$	(Ma et al., 2009b)
93	apigenin-7-O-β-D-glucopyranoside	$C_{21}H_{20}O_{10}$	(Ma et al., 2009a)
94	diosmetin7-O-β-D-glucopyranoside	$C_{22}H_{22}O_{11}$	(Ma et al., 2009b)
95	diosmetin	$C_{16}H_{12}O_{6}$	(Ma et al., 2009a)
96	alfalfa-7-O-β-D-glucopyranoside	————	(Ma et al., 2009b)
97	quercetin 3-O-β-D-glucopyranoside	$C_{21}H_{18}O_{13}$	(Ma et al., 2009a)
98	flavonolignan	————	(Wang et al., 2016)
99	eriodictyol	$C_{15}H_{12}O_{6}$	(Wang et al., 2016)
100	chrysoeriol	$C_{16}H_{12}O_{6}$	(Wang et al., 2016)
101	dioscorea grosvenorii biflavone A	$C_{31}H_{20}O_{10}$	(Wang et al., 2016)
102	cypress bisflavonoid	$C_{30}H_{18}O_{10}$	(Wang et al., 2016)

### 2.4 Triterpenoids and triterpene saponins

**FIGURE 4:**
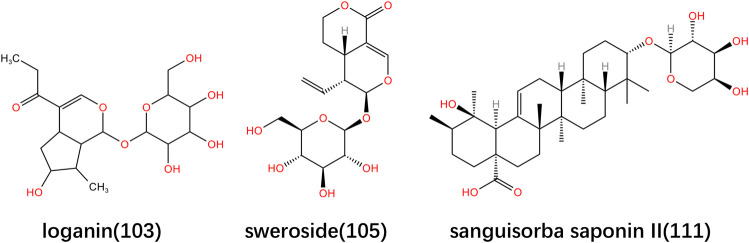
Structural formula of major triterpenoids and triterpene saponins in *Lonicerae Japonicae (Caulis)*.

*Lonicerae Japonicae Caulis* contains many terpene metabolites, including triterpenoids, triterpene saponins, etc. A large number of studies have shown that the terpenoids contained in *Lonicerae Japonicae (Caulis)* have anti-inflammatory and antitumor activities (Kwak et al., 2003; Li and Mu et al., 2017; Ge W. et al., 2018; Mei et al., 2019). As an essential triterpene saponin, aescin can significantly inhibits lung cancer cell proliferation by downregulating protein kinases such as AKT, mTOR, MEK, and ERK. It also can induce cytotoxic autophagy-mediated cell apoptosis withthe downregulation of AKT mTOR. In addition, aescin downregulates the expression of the HIF-1α and VEGF gene to reduce the migration and invasion ability of cells. Based on this, further research and exploration can be considered on the interaction between aescin and macromolecular compounds with migration ability in the future. In addition, the preclinical DEN-induced lung cancer model successfully monitored the expression of EGFR gene, improved the lung histology, and regulated the biochemical parameters. However, the above findings have not been further validated so far. So it is necessary to conduct the studies *in vivo* on xenograft or genetic animal models (Singh et al., 2023). Sweroside isolated from *Lonicera japonica* can regulate the expression of MAP kinase and melanogenesis enzyme, which may be an effective skin-whitening agent, which can be used as raw materials for cosmetics and fragrances (Jeong et al., 2015). The specific metabolite information is shown in Table 4, and the main metabolites structures are shown in Figure 4.

**TABLE 4:** Triterpenoids and triterpene saponins in *Lonicerae Japonicae (Caulis)*.

No.	Compound Name	Formula	Ref.
103	loganin	$C_{17}H_{26}O_{10}$	(Ma et al., 2010)
104	cyclosporin	————	(Mehrotra et al., 2004)
105	sweroside	$C_{16}H_{22}O_{9}$	(Ma et al., 2010)
106	cyclosporin dimethyl acetal	————	(Ma et al., 2011)
107	secologanin	————	(Ma et al., 2011)
108	deoxidized loganin	$C_{17}H_{22}O_{9}$	(Wang et al., 2016)
109	strychnoside semialdolactone	$C_{17}H_{24}O_{10}$	(Wang et al., 2016)
110	secologanin	$C_{17}H_{24}O_{10}$	(Wang et al., 2016)
111	sanguisorba saponin II	$C_{35}H_{56}O_{8}$	(Wang et al., 2016)
112	pine resin phenol 4-O-β-D-glucopyranoside	————	(Wang et al., 2016)
113	2-methoxyhydroquin-4-O-β-D-glucoside	————	(Ma et al., 2010)

### 2.5 Other metabolites

**FIGURE 5:**
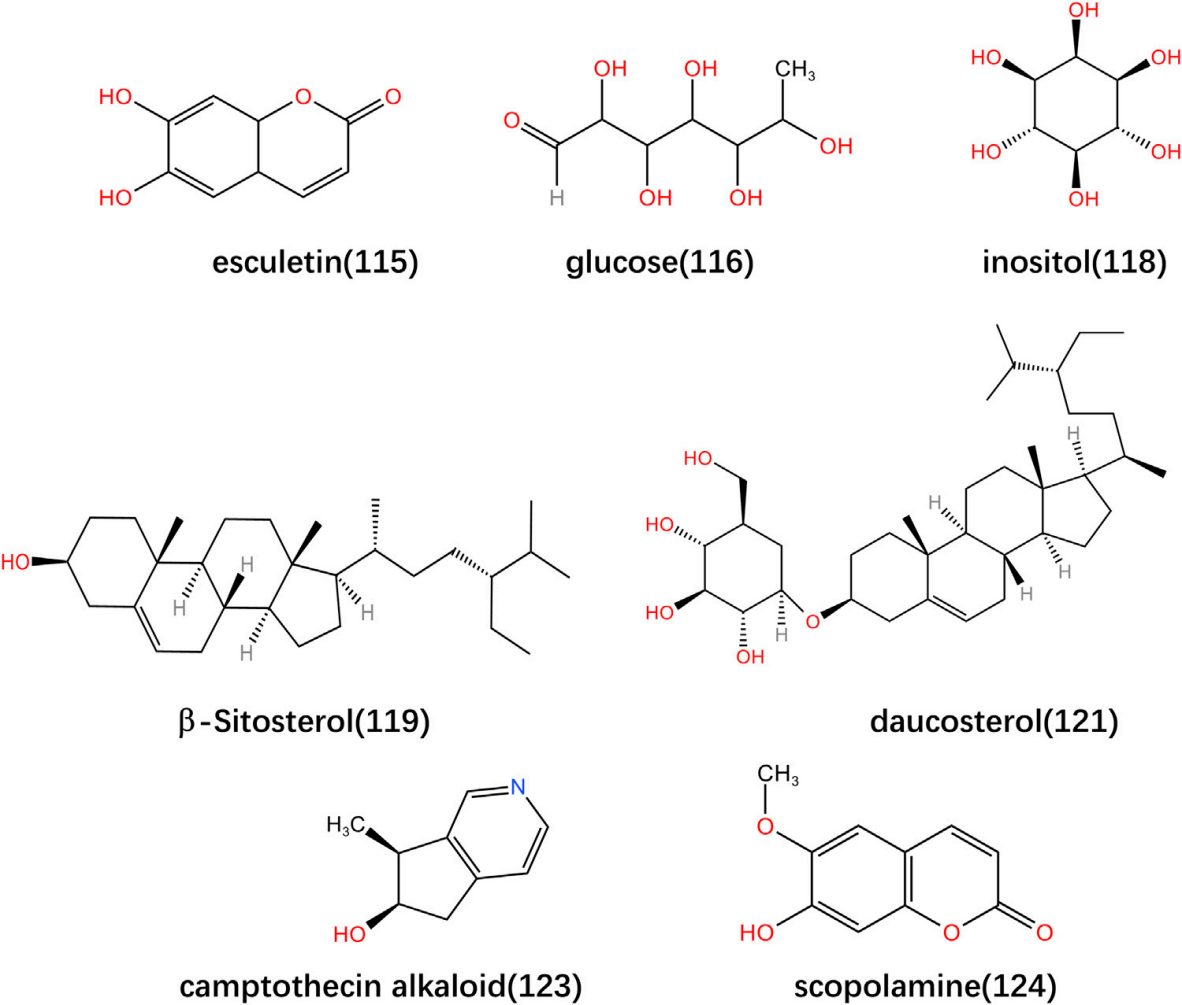
Structural formula of other main active ingredients in *Lonicerae Japonicae (Caulis)*.

The chemical metabolites of *Lonicerae Japonicae Caulis* are complex, diverse, and have multiple effects. In addition to the four main chemical substances mentioned above, it includes numerous other metabolites. Chen Ling et al. isolated inositol, uracil nucleoside, 2-methoxyhydroquinone 4-O- β- D-glucoside, and (+) turpentin-4-O- β- D-glucoside from *Lonicera japonica* vine (Chen et al., 2015). Expect the above active metabolites, *Lonicerae Japonicae (Caulis)* also contains some amino acids, such as Alanine (Ala), Serine (Ser), Proline (Pro), Valine (Val), Threonine (Thr), Isoleucine (Lle), Leucine (Leu), Aspartic acid (Asn), Glutamic acid (Gln), Lysine (Lys), Histidine (His), Phenylalanine (Phe), and Arginine (Arg), as well as trace elements such as Iron (Fe), Magnesium (Mg), Copper (Cu), Chromium (Cr), Manganese (Mn), Zinc (Zn), Nickel (Ni), Arsenic (Se), Molybdenum (Mu), Selenium (Xi), Cadmium (Cd), Mercury (Hg) and Lead (Pb) (Yu et al., 2013; Zhao et al., 2018; Cai et al., 2019). In addition, it contains four nucleosides, Cytidine, Uridine, Adenosine, and Inosine. The specific composition information is shown in Table 5, and the main metabolites structures are shown in Figure 5.

**TABLE 5:** The other active ingredients in *Lonicerae Japonicae (Caulis)*.

No.	Compound Name	Formula	Ref.
114	honeysuckle alcohol	$C_{10}H_{18}O_{2}$	(Zhao et al., 2007)
115	esculetin	$C_{9}H_{6}O_{4}$	(Zhang et al., 2009)
116	glucose	$C_{6}H_{12}O_{6}$	(Ma et al., 2010)
117	N-29 alcohol	$C_{29}H_{59}OH$	(Ma et al., 2010)
118	inositol	$C_{6}H_{12}O_{6}$	(Ma et al., 2010)
119	β-Sitosterol	$C_{29}H_{50}O$	(Wang et al., 2016)
120	β-Sitosterol glucoside	$C_{35}H_{60}O_{6}$	(Ma et al., 2010)
121	daucosterol	$C_{35}H_{60}O_{6}$	(Ma et al., 2010)
122	(22E, 24R)ergoste-7,22-diene-3 β, 5 α, 6 β- Triol	————	(Ma et al., 2010)
123	camptothecin alkaloid	$C_{9}H_{11}NO$	(Ma et al., 2010)
124	scopolamine	$C_{10}H_{8}O_{4}$	(Ma et al., 2010)

## 3 Pharmacological action and mechanism of *Lonicerae Japonicae Caulis*

The crude extract and the monomer metabolites contained in *Lonicerae Japonicae Caulis* have pharmacological effects such as the inhibition of pathogenic microorganisms, anti-rheumatism, anti-inflammatory, bone and soft tissue repair, antitumor, anti-oxidation, anti-allergic reaction, and immune regulation function.

### 3.1 Anti-pathogenic microbial effect

**FIGURE 6:**
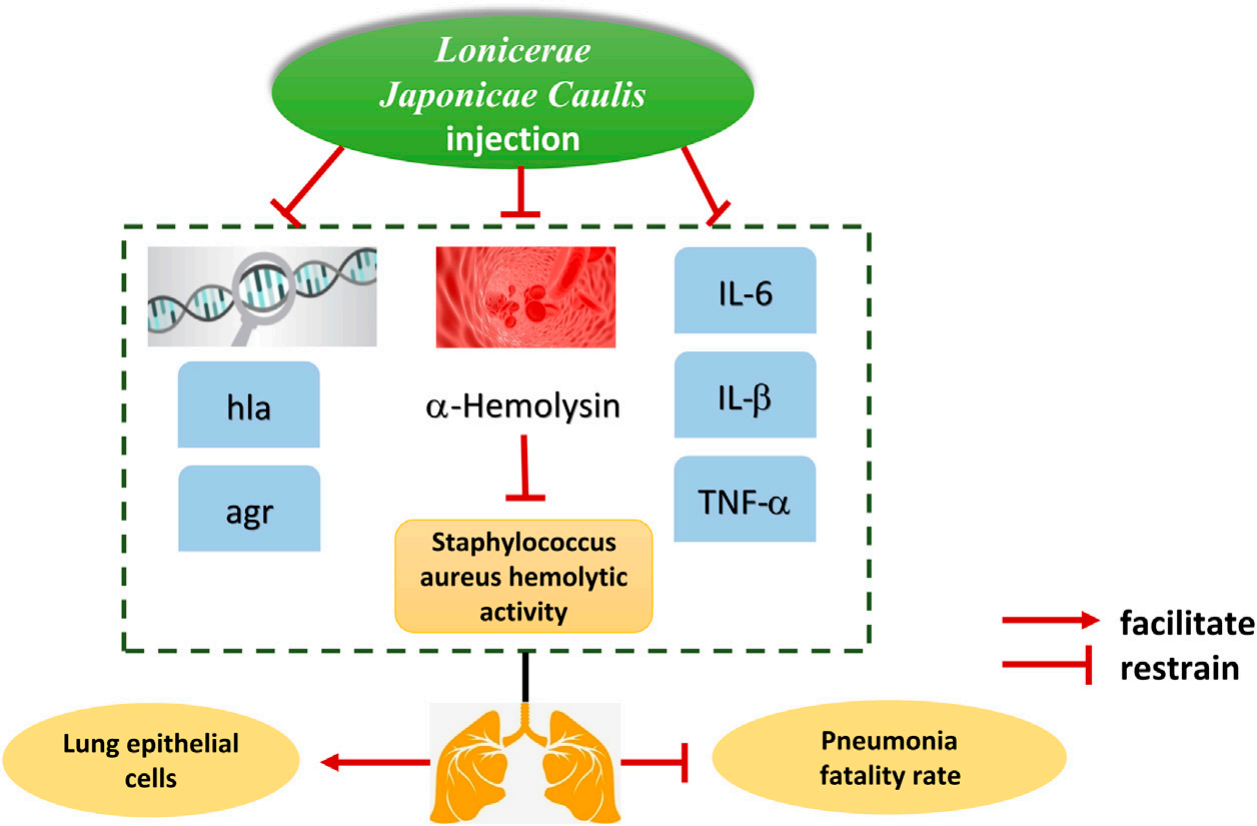
Protective mechanism of *Lonicerae Japonicae Caulis* lung epiphyteal cells in mice with *Staphylococcus aureus* pneumonia.

The main bacteriostatic active metabolites in *Lonicerae Japonicae Caulis* are chlorogenic acid and flavonoids, and the bacteriostatic effect of flavonoids is more significant than that of chlorogenic acid (Xiong et al., 2013; Yang et al., 2016; Minami and Makino, 2020; Yan et al., 2020). *Lonicerae Japonicae Flos* is superior to *Lonicerae Japonicae Caulis* in the prevention and treatment of respiratory diseases, and *Lonicerae Japonicae Caulis* is better than *Lonicerae Japonicae Flos* in the treatment of infectious hepatitis and infectious mumps (Zhao et al., 2016). 214 cases of chronic hepatitis B patients were collected, and the dialectical treatment with *Lonicerae Japonicae Caulis* powder was carried. The results showed that the level of endothelin (ET) in the treated patients decreased significantly, confirmed that *Lonicerae Japonicae Caulis* had the effect of repairing endothelial cells and had a specific effect on the chronic hepatitis B cases caused by hepatitis B virus (Zhou and Yu, 2002). Luo Mingjing found that *Lonicerae Japonicae Caulis* injection could reduce the expression level of α-hemolysin and its related regulatory genes, reduce the secretion of α-hemolysin, reduce the damage of *Staphylococcus aureus* to lung epithelial cells and reduce the fatality rate of *Staphylococcus aureus* pneumonia, suggesting that *Lonicerae Japonicae Caulis* injection has an inhibitory effect on *Staphylococcus aureus* (Figure 6) (Luo, 2012). This experiment analyzed the secretion of α-hemolysin and the mechanism of *Lonicerae Japonicae Caulis*’s anti-staphylococcus aureus pneumonia effect from the perspective of virulence factors. It laid the foundation for further exploring the complex mechanism of action of *Lonicerae Japonicae Caulis* in *staphylococcus aureus* pneumonia. Although the research has shown that *Lonicera japonica* injection has a significant therapeutic effect on pneumonia caused by *staphylococcus aureus*, relevant data has not yet been obtained in the clinical practice, and further research results on the clinical application need to be followed up. Zuo Huifen et al. compared the specific effects of *Lonicerae Japonicae Caulis*, *Plum* and *Lonicerae Japonicae Flos* on Steurophila maltophila by Mueller-Hinton Agar dilution method, and the results showed that they all had different degrees of bacteriostatic effects on this bacterium (Zuo et al., 2020). The specific antibacterial metabolites of the three traditional Chinese medicines against Stenotrophomonas maltohilia have not been identified here. This can be considered as the next research content. Chen Weiyan prepared the four kinds of extracts from the traditional Chinese Medicine of *Hemibranch*, *Mutong*, *Lonicerae Japonicae Caulis* and *Forsythia* by microwave, and analyzed their inhibitory effects on Citrus anthracnose, Cotton wilt and Wheat Total Eclipsing Bacteria (Chen, 2022). The results showed that *Lonicerae Japonicae Caulis* had a strong bacteriostatic effect under the extraction of ethanol, acetone and ethyl acetate, and the bacteriostatic effect could reach 100% under the condition of acetone solvent, which confirmed the excellent effect of *Lonicerae Japonicae Caulis* in inhibiting pathogenic microorganisms. Some studies have shown that *Lonicerae Japonicae* can significantly promotes the colonization of beneficial bacteria and inhibits the reproduction of harmful bacteria (Wang et al., 2014; Minami and Makino, 2020). The defense mechanisms against bacteria, such as the activation of neutrophils, lymphocytes and complement systems, require further research.

### 3.2 Anti-inflammatory effect

**FIGURE 7:**
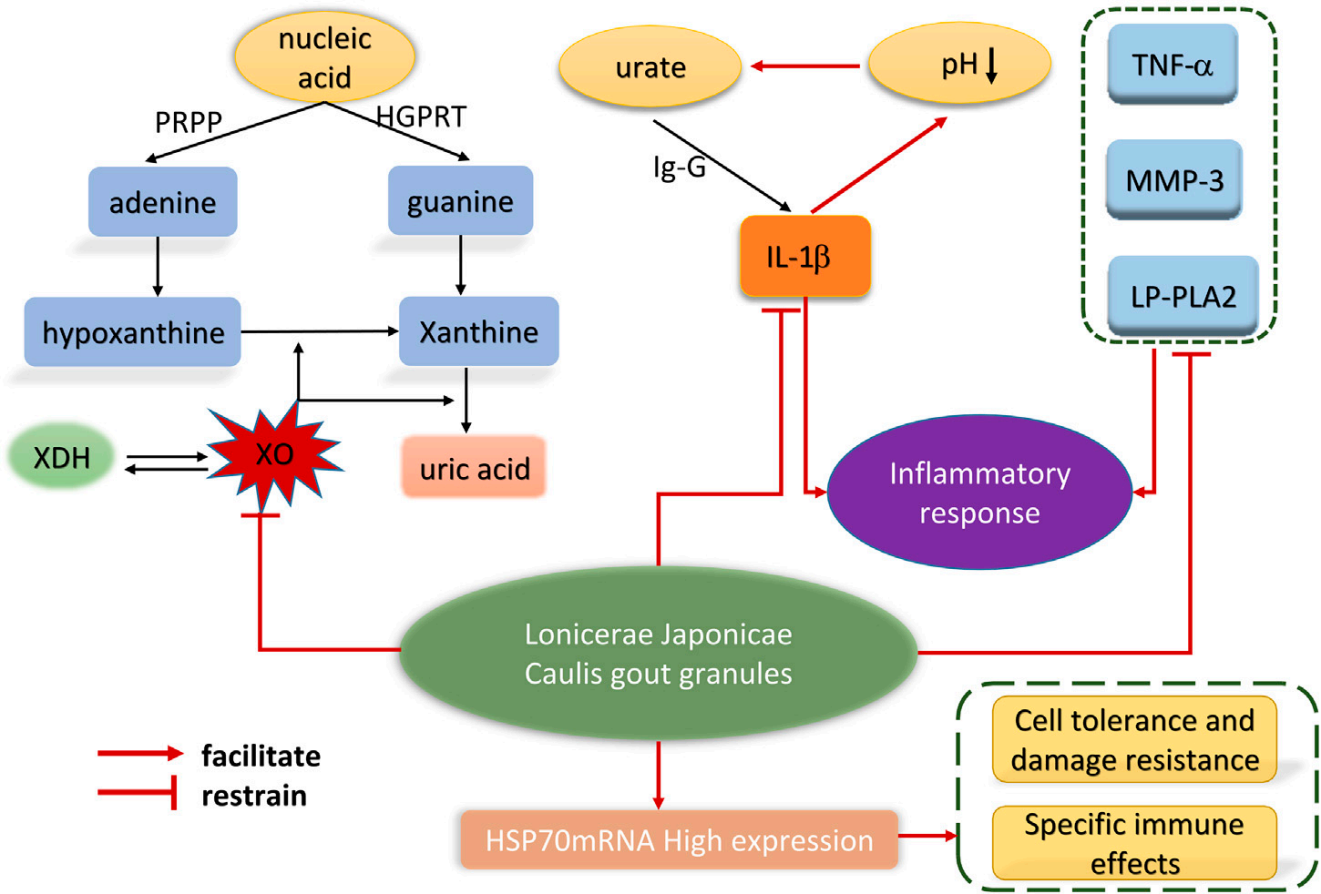
Mechanism of *Lonicerae Japonicae Caulis* granules in the treatment of gouty arthritis.

Inflammation refers to a normal protective response when the body is damaged and infected. In the research and response of traditional Chinese Medicine, *Lonicerae Japonicae (Caulis)* is often combined with other traditional Chinese Medicines to treat inflammation and has significant anti-inflammatory effects *in vivo* and *in vitro* (Jiang M. et al., 2014; Li N. et al., 2020; Zhou et al., 2021). Gout is an inflammatory joint disease, and studies have found that the use of fire-needle therapy for bloodletting and the oral *Lonicerae Japonicae Caulis* soup for the treatment of gout patients can improve the level of inflammatory factors, promote inflammatory absorption, and relieve pain (Zhao and Chen, 2019). Another study found that using *Lonicerae Japonicae Caulis* granules to intervene in gouty arthritis rats, the results showed that the blood uric acid (BUA) level, liver xanthine oxidase (XO) activity, interleukin-1β (IL-1β), matrix metallo proteinase-3 (MMP-3), lipoprotein phospholipase A2 (LP-PLA2) and tumor necrosis factor-α (TNF-α) levels in gouty arthritis rats were significantly reduced, confirming that *Lonicerae Japonicae Caulis* has anti-inflammatory and analgesic effects (Wang, 2016). The specific action mechanism is shown in Figure 7. For the sake of comparability of experimental data, male rats were used in this study. Considering the gender differences in clinical practice, this study lacks a certain degree of objectivity. Although the mechanism in treating gout is becoming clear increasingly, due to the complex metabolites contained in traditional Chinese medicine. It is difficult to observe a single variable and locate precise targets. Therefore, further exploration is needed for the deeper mechanism study. *Lonicerae Japonicae Caulis* decoction was used to treat patients with humid heat and the connotation of uric acid nephropathy. The observation symptoms showed that the symptoms of joint burning pain and lower limb edema were significantly reduced. Renal function was improved, and blood uric acid and blood β2 microglobulins were significantly reduced, indicating that *Lonicerae Japonicae Caulis* has anti-inflammatory activity and a specific effect on the humid heat connotation of uric acid nephropathy (Li R. J. et al., 2020). Chen Hening et al. used the water-extracted of Astragalus, Angelica and *Lonicerae Japonicae Caulis* freeze-dried powderon the rat synovial cells, observed and monitored the morphological changes and apoptosis rate of synovial cells, as well as the expression levels of criticalproteins in NF-κB and JAK/STAT pathways. The results showed that the extract might increase the apoptosis rate of synovial cells by inhibiting NF-κB and JAK/STAT signaling pathways, in the end ultimately inhibit the synovial cell proliferation and alleviate the inflammatory symptoms (Chen et al., 2020). The water decoction of *Astragalus*, *Angelica* and *Lonicerae Japonicae Caulis* dry powder were prepared respectively to intervene in inflammatory injury model rats by Meng Xiaoying. The results showed that the content of T-cells increased, the mobility decreased and the chemokine-2 (CCL2) increased. The mechanism was related to the inhibition of synovial cell inflammatory proliferation in rats, which positively affected the prevention and treatment of rheumatoid arthritis (Meng, 2021). Lan Huangqi et al. used *Litsea pungens Hemsl* and *Lonicerae Japonicae Caulis* extracts on asthma model mice and found that its mechanism of action was reducing the levels of IgE, IL-4, IL-5, and IL-10 contained in the serum, increase the level of IFN-γ, and weaken the expression of GATA-3 protein, confirming that *Lonicerae Japonicae Caulis* can be used to improve airdoritis in the asthmatic mice (Lan et al., 2020a). Dou Yuyu et al. used *Litsea pungens Hemsl* and *Lonicerae Japonicae Caulis* in asthma model mice based on the experimental research of Lan huangqi, and found that the drug could reduce the levels of IGF-β1, IL-4, IL-13, VEGF, PDGF and the expression levels of related proteins Cyclin D1 and ERK1/2, thereby inhibiting the proliferation of airway smooth muscle cells and achieving anti-inflammatory effects (Dou et al., 2021). Another study has shown that *Lonicera japonica* can exert anti-inflammatory effects on the LPS-induced lung inflammation, improve lung morphology, and reduce pulmonary edema. *Lonicerae Japonicae Caulis* increases the nuclear Sp1 binding activity through incremental phosphorylation of ERK, thereby enhancing IL-10 expression. Simultaneously, it can reduce nuclear NF-κB binding activity by suppressing the phosphorylation of IκB, p38, and JNK, thereby inhibiting the expression of TNF-α, IL-1β and IL-6 in the lungs (Kao et al., 2015). The above studies have shown that *Lonicerae Japonicae Caulis* can play a good role in treating inflammatory diseases. However, further research is needed to confirm the specific effects of natural metabolites contained in *Lonicerae Japonicae Caulis* on macrophages under *in vitro* conditions.

### 3.3 Repair bone and soft tissue

**FIGURE 8:**
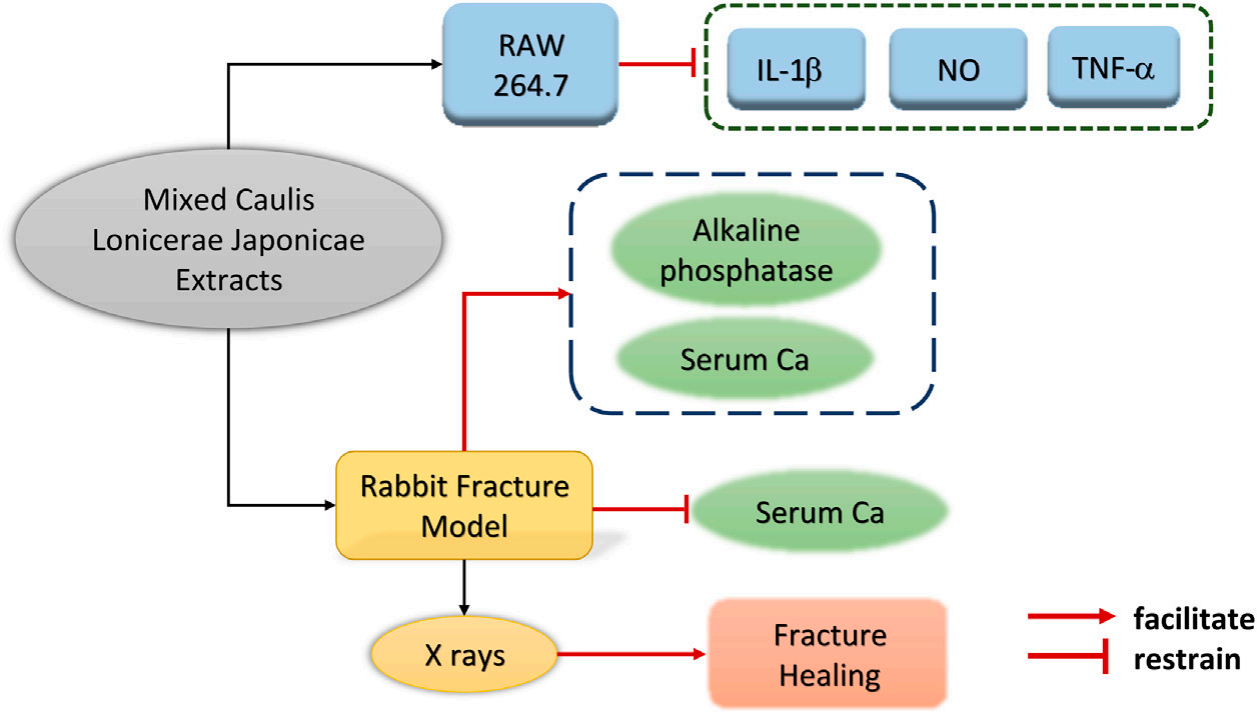
Mechanism of effects of Mixed *Caulis Lonicerae Japonicae* Extracts on Fracture Healing and Anti-inflammation.

Modern research has shown that *Lonicerae Japonicae Caulis* has a definite role in the repairing of the bone and soft tissue. Jia Haiyan found that *Lonicerae Japonicae Caulis* had a significant effect on the content of calcium and alkaline phosphatase in the serum of fractured rabbits, and had a positive effect on the repair of fractures (Jia et al., 2017). The specific mechanism of action is shown in Figure 8. Huang Xin used a self-formulated prescription including *Rhubarb* (30 g), *Bitter ginseng* (30 g), *Lonicerae Japonicae Caulis* (30 g), and *Bai Zhi* (20 g) to treat soft tissue sprain patients (Huang, 1995), and the results showed that the total effective rate was as high as 97% in 200 patients, which proved that *Lonicerae Japonicae Caulis* had a good effect on dissipating local bruising and repairing soft tissues. In addition, *Lonicerae Japonicae Caulis* is effective in treating chronic osteomyelitis (COM). In summary, *Lonicerae Japonicae Caulis* positively can positively indeed promote the repair of bone and soft tissue.

### 3.4 Antitumor effect

**FIGURE 9:**
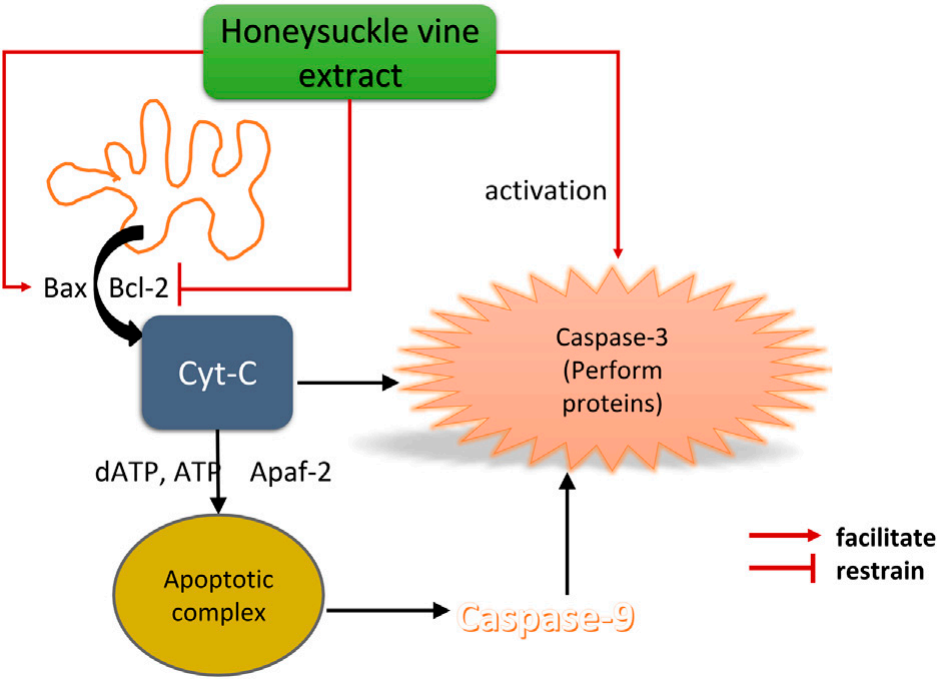
Mechanism of *Lonicerae Japonicae Caulis* Rattan extract inducing apoptosis of osteosarcoma cells.

The flavonoids and saponins in *Lonicerae Japonicae Caulis* also have certain antitumor pharmacological effects. Shan Yu et al. found that macranthoside B (MB), a saponin metabolite, could block the proliferation of human ovarian cancer A2780 cells and induce apoptosis and autophagy (Shan et al., 2016). Yan Baofei et al. found that *Lonicerae Japonicae Caulis* could achieve the purpose of apoptosis of human osteosarcoma cells (HOS) through mitochondrial apoptosis (Yan et al., 2021). *Lonicerae Japonicae Caulis* extract can reduce the mitochondrial membrane potential and Bcl-2 protein expression levels of HOS, and increase the expression level of Bax and cleaved Caspase-9 protein, thereby inhibiting the survival rate of HOS and 143B cells. The specific mechanism were shown in Figure 9. This study conduct a comprehensive and rapid qualitative analysis of the chemical metabolites of *Lonicerae Japonicae Caulis* based on UPLC-Q-TOF/MS. In the later study, it can be considered to start further elucidating the anti-tumor mechanism of *Lonicerae Japonicae Caulis* with the interaction between specific metabolites. There are also studies using the rough alcohol extraction of *Lonicerae Japonicae Caulis* extraction to carry out tumor suppression experiments *in vivo* and tumor-killing experiments *in vitro*. Its tumor inhibition rate is 30% higher, which confirms the antitumor effect of Lonicerae Japonicae Caulis (Li et al., 2000). Chen Ling et al. used the *Lonicerae Japonicae Caulis* extract on the colon cancer cells. Some Researchers detected the proliferation of colon cancer cells after medication and tested the change of mitochondrial membrane potential of cancer cells. Besides, the expression level of cell-related proteins was measured. Some researchers detected the effects of *Lonicerae Japonicae Caulis* extract and a kind of p53 inhibitor (PFT-α) on the apoptosis of cancer cells. The results showed that *Lonicerae Japonicae Caulis* extract could induce apoptosis in p53-dependent mitochondria and had the anti-colon cancer effects (Chen et al., 2022). Researchers isolated and identified four flavonoids from *Lonicerae Japonicae*, all of which exhibited anti-liver cancer activity *in vitro* experiments and had a protective effect on the liver. At present, in the clinical treatment of liver cancer, the common chemotherapy drugs doxorubicin (Dox), lipiodol, and cisplatin all have serious adverse reactions (Ge L. L. et al., 2018). The protective effects of flavonoids from the flowers, leaves, and stems of *Lonicera japonica* on liver cancer may indicate the potentiality of *Lonicerae Japonicae Caulis* as being a health food resource during chemotherapy for liver cancer.

### 3.5 Antioxidant effect

The antioxidant effect is one of the important biological activities of *Lonicerae Japonicae (Caulis)* (Hus et al., 2016; Lee et al., 2019; Wan et al., 2019; Zhang et al., 2020). Guo et al. (2014) found that *Lonicerae Japonicae Caulis* had antioxidant activity *in vivo* and *in vitro*. Its antioxidant activity is closely related to the polyphenols and polysaccharides rich in *Lonicerae Japonicae*, including various phenolic acids and flavonoid metabolites (Guo et al., 2014; Fan et al., 2019). Xiang Jiqian et al. used 3 micro-models of 1,1-diphenyl-2-picrylhydrazyl (DPPH), 2,2'-azino-bis (3-ethylbenzothiazoline-6-sulfonic acid) diammonium salt (ABTS) and ferric reducing antioxidant power (FRAP) as indicators to detect the effects of different flavonoid extraction processes on the extraction of total flavonoids in *Lonicerae Japonicae Caulis* and their antioxidant effect *in vitro* (Xiang et al., 2017). The results showed that total flavonoids of *Lonicerae Japonicae Caulis* had high antioxidant capacity *in vitro*. Chlorogenic acid (CGA) and caffeic acid (CA), which belong to phenolic acids, have significant antioxidant activity *in vitro* and *in vivo* and are potent antioxidants (Wang et al., 2009; Li and Wang, 2012; Jiang P. et al., 2014). Chen Liangmian et al. used DPPH, ABTS and FRAP to determine that *Lonicerae Japonicae Caulis*, *Lonicerae Japonicae Flos* and *Folium Lonicerae* all had different degrees of antioxidant effects (Chen et al., 2021). In addition, the anthocyanins rich in *Lonicerae Japonicae Caulis* have significant anti-inflammatory and antioxidant activities. Denis Golubev et al. studied the antioxidant and anti-aging activities of *Lonicerae Japonicae* extract with the model of *drosophila melanogaster*. The results showed that for male and female fruit flies, supplementation with 100 uM concentration of *Lonicerae Japonicae* extract (LE) and anthocyanins could better improve the life span of fruit flies (Golubev et al., 2022).

### 3.6 Immunomodulatory effect

*Lonicerae Japonicae Caulis* also has a regulatory effect on the body’s immune function. Meng Xiaoying used *Lonicerae Japonicae Caulis*, *Astragalus* and *Angelica* to regulate the T-cell immune system, thereby inhibiting the expression of CCL2 and further reducing the inflammatory response of the rat synovial cells (Meng et al., 2019). Lan Huangqi et al. applied the ethanol extract of Litsea pungens Hemsl and *Lonicerae Japonicae Caulis* to treat the asthma model mice. It was found that the levels of IgE, IL-4, IL-5, and IL-10 in mice were significantly reduced, while IFN- γ level significantly increased and the expression level of GATA-3 protein was also improved. The above results suggest that *Lonicerae Japonicae Caulis* has a regulatory effect on the immune function in the body. But there is currently no specific explanation for its upstream mechanism of regulating GATA-3 expression (Lan et al., 2020b). In addition, the flavonoid substances in *Lonicerae Japonicae Flos* can enhance immunity by removing superoxide ion free radicals in the body (Yang et al., 2017). *Lonicerae Japonicae Caulis* is widely used in clinical applications. *Lonicerae Japonicae Caulis* decoction affects immune infertility caused by anti-sperm antibody (AsAb) positive (Ma et al., 2003). And *Lonicerae Japonicae Caulis* can be combined with *Caulis spatholobi* to treat the autoimmune diseases, such as systemic lupus erythematosus, allergic purpura, and thrombocytopenic purpura, etc. Besides, it has achieved good clinical efficacy, and the addition of *Lonicerae Japonicae Caulis* to broiler feed can improve the conversion rate of broiler feed, increase the phagocytosis of broiler thymus, spleen index, and macrophages (Wu et al., 2007; Sun et al., 2010). It is also suggested that *Lonicerae Japonicae Caulis* can enhance the immune function of the animal body.

## 4 Summary and research prospect

*Lonicerae Japonicae Caulis* has the effect of clearing away heat, toxic material, dredging wind and unblocking collaterals in the theory of traditional Chinese Medicine. It is mainly used clinically for the prevention and treatment of warm fever. Its various traditional uses have now been confirmed by modern pharmacology, and its various active metabolites have been confirmed by some researches. Due to the abundant content and pharmacological effects of the active metabolite CGA, it has been used as a marker to characterize the chemical properties of *Lonicerae Japonicae Caulis*, and *Lonicerae Japonicae Flos*. Still, CGA is not specific, and further research is needed to determine whether the quality of the drug is entirely appropriate when used to determine whether the quality is entirely appropriate.

It is precisely because of the various pharmacological activities of *Lonicerae Japonicae Caulis* that it plays an increasingly important role in treating various a variety of diseases. At the same time, because *Lonicerae Japonicae Caulis* belongs to the homologous resources of medicine and food, and it has the characteristics of medicine and food. It has natural advantages in safety and tonics, so the public loves it. In addition, the research and exploration of the pharmacological activity of *Lonicerae Japonicae Caulis* can also help develop and prepare new drugs from *Lonicerae Japonicae Caulis*. In recent years, many studies have been on the chemical metabolites and pharmacological activities of *Lonicerae Japonicae Caulis*. However, it is unclear whether there are interactions between the various chemical metabolites. In addition, the impact of environmental factors on the types and contents of chemical metabolites that *Lonicerae Japonicae Caulis* contains is currently not fully understood. The differences in the effect mechanisms of various single metabolites and metabolites groups on the various diseases have not been fully explored and studied. It is possible to explore the pharmacological effects of classic prescriptions. Although many studies have confirmed the various pharmacological activities of *Lonicera japonica* vine, there is still a lack of research on its clinical application. Therefore, in future research and exploration, the scope of material foundation research and the depth of pharmacological activity research should be expanded, and clinical practice research should be emphasized.

In summary, the active metabolites contained in *Lonicerae Japonicae Caulis* are complex, the pharmacological effects are diverse. It has a positive effect in the prevention and treatment of respiratory diseases, cancer, cardiovascular and cerebrovascular diseases, liver diseases, etc., which confirms that the potential application value of *Lonicerae Japonicae Caulis* is outstanding, which is worthy of follow-up in-depth research.

## Abbreviations

AsAb, Anti-sperm Antibody; BUA, Blood Uric Acid; CCL2, Chemokine-2; COM, Chronic osteomyelitis; CA, Caffeic Acid; CGA, Chlorogenic Acid; ET, Endothelin; HOS, Human osteosarcoma cell; IL-1β, Interleukin-1β; LE, *Lonicerae Japonicae Caulis* extract; LP-PLA2, Lipoprotein Phospholipase A2; LPS, Limpopolysaccharide; MMP-3, Matrix Metalloproteinase-3; MB, Macranthoside B; TNF-α, Tumor Necrosis Factor-α; XO, Xanthine oxidase.
